# Metabolomics reveals distinct neurochemical profiles associated with stress resilience

**DOI:** 10.1016/j.ynstr.2017.08.001

**Published:** 2017-08-07

**Authors:** Brooke N. Dulka, Allen K. Bourdon, Catherine T. Clinard, Mohan B.K. Muvvala, Shawn R. Campagna, Matthew A. Cooper

**Affiliations:** a Department of Psychology, University of Tennessee, Knoxville, TN 37996, United States; b Department of Chemistry, University of Tennessee, Knoxville, TN 37996, United States; c Biological Small Molecule Mass Spectrometry Core, University of Tennessee, Knoxville, TN 37996, United States

**Keywords:** Metabolomics, Biomarkers, Resilience, Stress, Oxidative stress, Social defeat

## Abstract

Acute social defeat represents a naturalistic form of conditioned fear and is an excellent model in which to investigate the biological basis of stress resilience. While there is growing interest in identifying biomarkers of stress resilience, until recently, it has not been feasible to associate levels of large numbers of neurochemicals and metabolites to stress-related phenotypes. The objective of the present study was to use an untargeted metabolomics approach to identify known and unknown neurochemicals in select brain regions that distinguish susceptible and resistant individuals in two rodent models of acute social defeat. In the first experiment, male mice were first phenotyped as resistant or susceptible. Then, mice were subjected to acute social defeat, and tissues were immediately collected from the ventromedial prefrontal cortex (vmPFC), basolateral/central amygdala (BLA/CeA), nucleus accumbens (NAc), and dorsal hippocampus (dHPC). Ultra-high performance liquid chromatography coupled with high resolution mass spectrometry (UPLC-HRMS) was used for the detection of water-soluble neurochemicals. In the second experiment, male Syrian hamsters were paired in daily agonistic encounters for 2 weeks, during which they formed stable dominant-subordinate relationships. Then, 24 h after the last dominance encounter, animals were exposed to acute social defeat stress. Immediately after social defeat, tissue was collected from the vmPFC, BLA/CeA, NAc, and dHPC for analysis using UPLC-HRMS. Although no single biomarker characterized stress-related phenotypes in both species, commonalities were found. For instance, in both model systems, animals resistant to social defeat stress also show increased concentration of molecules to protect against oxidative stress in the NAc and vmPFC. Additionally, in both mice and hamsters, unidentified spectral features were preliminarily annotated as potential targets for future experiments. Overall, these findings suggest that a metabolomics approach can identify functional groups of neurochemicals that may serve as novel targets for the diagnosis, treatment, or prevention of stress-related mental illness.

## 1 Introduction

Stress is a contributing factor in the etiology of several psychiatric conditions including depression (Heim et al., 2008), panic disorder (Abelson et al., 2007), and post-traumatic stress disorder (PTSD) (Meewisse et al., 2007). Aggression is a particularly salient form of trauma, and people exposed to interpersonal violence are at a greater risk for developing PTSD than those exposed to non-personal trauma (Charuvastra and Cloitre, 2008). However, many individuals who experience stressful events do not develop a stress-related psychopathology, and there is a great deal of interest in what makes certain individuals resilient. Stress resilience refers to the ability of individuals to maintain normal levels of psychological, biological, and social functioning following a traumatic event. Importantly, resilience is an active process and not simply the absence of a pathological response to stress (Charney, 2004, Russo et al., 2012, Feder et al., 2009).

Animal models of social defeat stress have been put forth as high validity models of stress-related mental illness and, interestingly, individuals exhibit pronounced variability to the effects of social defeat (Nestler and Hyman, 2010). Genetically identical, inbred mice display a great deal of variability in social avoidance following 10 days of chronic social defeat (Krishnan et al., 2007, Berton et al., 2006) and two days of repeated social defeat (Meduri et al., 2013, Dulka et al., 2015). Susceptible mice avoid novel animals in a social interaction test following social defeat stress, whereas resilient (or resistant) mice investigate novel animals following social defeat stress in a pattern similar to non-defeated controls (Golden et al., 2011). In the chronic social defeat model, brain-derived neurotrophic factor (BDNF) signaling in a neural circuit involving the ventral tegmental area and nucleus accumbens (NAc) is critical for the expression of defeat-induced social avoidance in susceptible animals (Berton et al., 2006). The ventromedial prefrontal cortex (vmPFC) also provides top-down inhibitory control of the NAc and amygdala, which promotes a resistant phenotype after social defeat stress (Vialou et al., 2014). In a mouse model using a single day of acute social defeat, BDNF signaling in the basolateral amygdala (BLA) is necessary for acquisition of defeat-induced social avoidance (Dulka et al., 2016). The development of the susceptible and resistant phenotypes is largely unknown, although the epigenetic changes that underlie stress vulnerability may be linked to environmental influences during pre-natal and post-natal development, including the establishment of early dominance hierarchies (Peaston and Whitelaw, 2006, Wong et al., 2005).

Syrian hamsters are aggressive and territorial animals that exhibit a striking change in behavior following social defeat stress. Following exposure to a single bout of social defeat, male hamsters fail to defend their home territory and instead exhibit submissive and defensive behavior toward novel non-aggressive intruders for up to one month (Huhman et al., 2003). This stress-induced change in agonistic behavior is called the conditioned defeat response and is similar to the defeat-induced social avoidance shown by rats and mice (Kudryavtseva, 1994, Meerlo et al., 1996). The conditioned defeat response in hamsters is an ethologically relevant form of conditioned fear and is regulated by many of the same brain regions, neural circuits, and neurochemicals as conditioned fear. Neurotransmission in the central amygdala (CeA) is critical for the expression of the conditioned defeat response (Jasnow and Huhman, 2001). In the BLA, NMDA receptors, BDNF, and cAMP response element binding (CREB) protein are each necessary for the acquisition of the conditioned defeat response (Day et al., 2011, Jasnow et al., 2005, Taylor et al., 2001). Neurotransmission in several other brain regions is known to modulate the conditioned defeat response, such as the NAc, ventral hippocampus (vHPC), and vmPFC (Gray et al., 2015, Markham et al., 2010, Markham et al., 2012). A great deal of variation exists in the amount of submissive and defensive behavior exhibited by hamsters following social defeat. To investigate vulnerability to the conditioned defeat response, we allowed dyads of hamsters to establish and maintain dominance relationships and then tested dominant and subordinate animals for their conditioned defeat response. We found that dominant hamsters show a reduced conditioned defeat response and increased c-Fos immunoreactivity in the vmPFC compared to subordinate and control animals (Morrison et al., 2011, Morrison et al., 2014). Furthermore, pharmacological blockade of neural activity in the vmPFC reinstated the conditioned defeat response in dominant hamsters but did not alter conditioned defeat in subordinates or controls (Morrison et al., 2013). While a great deal is known about the brain regions and neural circuitry that control the conditioned defeat response, relatively little is known about the neurochemistry within these structures.

There is growing interest in identifying neurochemical biomarkers to aid in the diagnosis, risk assessment, and prevention of stress-related mental illnesses such as PTSD (Yehuda et al., 2013, Zoladz and Diamond, 2013, Baker et al., 2012). Additionally, neurochemicals identified after a stressor can serve as mechanistic biomarkers, and such biomarkers can be used to improve the treatment of stress-related psychopathologies. While attempts to identify biomarkers continue to be a major focus of biomedical research, at present biomarkers have not made it into clinical application for mental illness. Part of the difficulty is that individual neurochemicals are unlikely to correlate with diagnosis, risk, or treatment response for complex forms of stress-related psychopathology. Taking a multifactorial approach is an essential first step toward developing biomarkers for mental illness. Metabolomics is a quantitative analysis of small molecules present in biological systems and has been increasingly used for the discovery of biomarkers (Griffiths et al., 2007, Oldiges et al., 2007, Kaddurah-Daouk et al., 2008). The use of untargeted metabolomics allows the user to take a discovery-based approach, which initially results in a data generating experiment. After relative quantitation of known metabolites based on pre-determined retention times and accurate mass (<5 ppm), the user is still left with thousands of unidentified spectral features (USFs) that potentially relate to a novel compound.

This study focused on characterizing the neurochemical profiles in select brain regions that distinguish animals that are susceptible and resistant to the effects of acute social defeat stress. In both mice and hamster models, we expected that susceptible and resistant animals would differentially express specific neurochemical metabolites in brain regions known to modulate defeat-induced changes in behavior. Further, a comparative approach is expected to aid in the discovery of biomarkers by identifying similar classes of compounds associated with stress susceptibility and resilience in both animal models.

## 2 Methods

### 2.1 Animals and housing conditions

Male C57BL/6 mice (7–8 weeks old, 20–27 g) were used as subjects (Envigo, Indianapolis, IN). Mice were maintained on a 12:12 light/dark cycle with *ad libitum* access to food and water in a temperature controlled room (21 ± 2 °C). Animals were housed in polycarbonate cages (18.4 cm × 29.2 cm x 12.7 cm) with corncob bedding, cotton nesting materials, and wire mesh tops. All behavioral procedures were performed during the first three hours of the dark phase of their cycle. Subjects were handled several times one week prior to social defeat to habituate them to the stress of human handling.

Male Syrian hamsters (3–4 months old, 120–180 g) were obtained from our breeding colony that is derived from animals purchased from Charles River Laboratories (Wilmington, MA). All animals were housed in polycarbonate cages (12 cm × 27 cm × 16 cm) with corncob bedding, cotton nesting materials, and wire mesh tops. Food and water were available *ad libitum*. Cages were not changed for one week prior to dominant–subordinate encounters to allow individuals to scent mark their territory. Subjects were handled several times one week prior to dominant–subordinate encounters to habituate them to the stress of human handling. Animals were housed in a temperature controlled colony room (21 ± 2 °C) and kept on a 14:10 h light:dark cycle to facilitate reproductive maturation. All behavioral protocols were performed during the first 3 h of the dark phase of their cycle. Procedures in both mice and hamsters were approved by the University of Tennessee Institutional Animal Care and Use Committee and are in accordance with the National Institutes of Health Guide for the Care and Use of Laboratory Animals.

### 2.2 Experimental procedures

#### 2.2.1 Social defeat stress

Mice were subjected to acute social defeat stress using a resident-intruder model adapted from the social defeat literature in Syrian hamsters (Huhman et al., 2003, McCann et al., 2014, Clinard et al., 2015). During social defeat stress, subjects were exposed to adult male Hsd:ICR (CD1) mice that were individually housed to maintain aggression (35–40 g, Evigo). The CD1 mice were prescreened for high levels of aggression, and animals exhibited attack latencies of less than 30 s. Social defeat stress consisted of three, 2-min aggressive encounters in the home cage of a novel CD1 resident aggressor mouse with 2-min inter-trial intervals in the subjects’ home cage, for a total duration of 10-min. These defeats were evaluated by an observer in real time to verify that each subject received multiple attacks from each CD1 mouse. An attack was defined as a rapid lunge followed by a bite or bite attempt. To correct for potential variation in the amount of aggression subjects received, the first defeat episode did not begin until the subject submitted to an attack from the resident aggressor. Non-defeated control animals were exposed to the empty home cage of three separate CD1 mice for 2-min with 2-min inter-trial intervals in their home cage.

In hamsters, social defeat stress consisted of subjects being placed in the home cages of three separate hamsters that were larger, older animals (>6 months, >190 g). These larger animals were called resident aggressors, and they were individually housed to maximize territorial aggression. Resident aggressors were also prescreened to ensure that they reliably attacked and defeated intruders. Subjects were exposed to three resident aggressors in consecutive 5-min aggressive encounters, with 5-min inter-trial intervals in their own home cage, for a total duration of 25-min. Similarly to mice, the first defeat episode did not begin until the subject submitted to an attack from the resident aggressor. Subjects submitted immediately in the second and third defeat episodes. Non-defeated control animals were placed in the empty home cages of three separate resident aggressors for three 5-min exposures to control for the novel environment and olfactory cues associated with social defeat stress. Social defeats were digitally recorded for behavioral analysis. The frequency of attacks by the resident aggressor was recorded and scored by a blind observer. Whether or not subjects fought back against the resident aggressor during the first social defeat episode was also recorded.

In both mice and hamsters, aggressive encounters were carefully monitored for wounding and animals that received minor scratches were treated with an antiseptic solution. No animal received a wound that resulted in signs of pain or distress and none of the animals were removed from the study because of wounding.

#### 2.2.2 Social interaction testing

Social interaction testing was performed in mice to identify animals that were susceptible and resistant to the effects of social defeat stress. Testing was performed in an open field arena (43.2 cm × 43.2 cm x 43.2 cm) under dim light conditions. Social interaction testing was modeled after conditioned defeat testing in Syrian hamsters (Huhman et al., 2003, McCann et al., 2014), as well as social interaction testing following chronic social defeat in mice (Golden et al., 2011). Social interaction testing consisted of two 5-min trials: CD1 mouse target absent and CD1 mouse target present. During the target absent trial, subjects were habituated to an empty perforated plastic box that was positioned against one of the four walls. The target present trial occurred immediately following the target absent trial, and a novel CD1 mouse was placed inside the perforated plastic box. The perforated box allowed for sensory information, but no physical contact. An observer blind to experimental conditions quantified the duration of time the subject spent in the interaction zone. The interaction zone was defined as a 3 cm area surrounding the plastic box. Considerable variation exists in behavioral responses to social defeat in C57 mice, and an interaction ratio has been used to categorize mice as either susceptible or resistant to social defeat (Krishnan et al., 2007, Golden et al., 2011). The interaction ratio is calculated as (Time investigating target present)/(Time investigating target absent). Defeated mice with interaction ratios of less than 1.0 are defined as susceptible and defeated mice with interaction ratios equal to or greater than 1.0 are defined as resistant to social defeat.

#### 2.2.3 Dominant-subordinate relationships

Hamsters were not exposed to social interaction testing because it was not necessary to phenotype animals following social defeat stress. Rather, hamsters were given the opportunity to establish dominance relationships, and we have previously shown that over time dominant animals become resistant to social defeat stress whereas subordinates become susceptible (Morrison et al., 2014). To establish social status, subjects were weight-matched into resident-intruder dyads and paired in daily social encounters for 14 days as described previously (Morrison et al., 2014). Subjects were randomly assigned as a resident or intruder, and all social encounters occurred in the resident's home cage. Encounters were 10-min in duration prior to the establishment of dominance relationships, while all subsequent encounters were 5-min. Dominant and subordinate animals were identified by the direction of agonistic behavior within each dyad. If a dyad did not form a dominance relationship after 5 encounters, the animals were excluded from statistical analysis.

#### 2.2.4 Experimental design

In Experiment 1, 62 mice were exposed to social defeat stress and 12 mice were non-defeated controls. Twenty-four hours later, mice received social interaction testing and were identified as susceptible (N = 51) or resistant (N = 11). Mice were tested until the resistant group reached sufficient statistical power, although brain tissue was collected from only the first 11 mice identified as susceptible. It is noteworthy that the percentage of resistant mice observed in this study (about 18%) is less than what has been reported elsewhere in chronic social defeat models (about 30%) (Krishnan et al., 2007). To investigate defeat-induced changes in neurochemical activity, mice received a second bout of social defeat stress or empty cage exposure one week following their social interaction test.

In Experiment 2, 30 hamsters were paired in daily dominant-subordinate encounters. Three dyads were excluded from analysis because they failed to establish a stable dominance relationship. Twenty-four hours after the final dominance encounter, 12 dominants and 12 subordinates received social defeat stress. Fourteen control animals did not receive dominance encounters and were exposed to empty cages instead of social defeat.

#### 2.2.5 Tissue collection

Immediately following the final social defeat encounter, animals were sacrificed with isoflurane and rapidly decapitated. A brain matrix was used to generate 1 mm thick brain slices that were rapidly frozen on glass slides. Tissue punches (1 mm diameter) were collected bilaterally from regions containing the BLA/CeA, dorsal hippocampus (dHPC), NAc, and vmPFC. Tissue punches were flash frozen in liquid nitrogen and stored at −80 °C until metabolite extraction. In some brain regions, tissue was not assayed because of inaccurate punches. In mice, 3 samples were lost from the BLA/CeA, 4 samples were lost from the dHPC, 2 samples were lost from the vmPFC, and 1 sample was lost from the NAc. In hamsters, due to inaccurate punches, 2 samples were lost from the BLA/CeA, 8 samples were lost from the dHPC, 7 samples were lost from the vmPFC, and 6 samples were lost from the NAc.

### 2.3 Untargeted metabolomics using ultra-high performance liquid chromatography-high-resolution mass spectrometry (UPLC-HRMS)

Analysis by UPLC-HRMS was completed in accordance with previously published protocols (Lu et al., 2010). Refer to Supplemental Information for additional details on the extraction protocol, chromatography specifications, and mass spectrometry (MS) parameters. The Metabolomic Analysis and Visualization Engine (MAVEN) software package, an open source program that reduces the complexity of metabolomics analysis, was used to select metabolites from a pre-existing list of over 270 compounds (Melamud et al., 2010). Henceforth, identified metabolites are referred to as known metabolites, which were previously validated for exact mass and retention time using chemical standards from Fisher Scientific or preexisting chromatography (Lu et al., 2010). MAVEN enables the user to directly extract all spectral features from a single sample. Any spectral feature that was not confirmed as a known metabolite will henceforth be referred to as an unidentified spectral feature (USF). Metabolite ion counts were normalized using the ratios of intensities among metabolites within a sample to remove errors due to sampling and instrumental variability using a modification of known methods (Altmaier et al., 2008, Suhre et al., 2011). This internal ratio normalization (IRN; see Supplemental Information) technique provides normalized fold changes for the selected array comparisons, as well as p-values from complimentary univariate analysis techniques, one-way ANOVA with Tukey post-hocs and two-tailed Student's t-tests. These fold changes were then used to generate heatmaps of known metabolites (for mice see Fig. S-1, for hamsters see Fig. S-2).

Additionally, unnormalized ion intensities of known and unknown metabolites were also analyzed using partial least squares discriminant analyses (PLS-DA). PLS-DA is a statistical method, similar to principal components regression, which finds a linear regression model by projecting predicated variables and the observed variables into a new space. From these PLS-DA analyses, variable importance in projection (VIP) score plots can be extrapolated. The VIP score is a common feature selection tool that is a probability function calculated by totaling the weighted sum of squares of the loading vectors (Xia et al., 2009), and this tool enables a matrix reduction of selected metabolites for further investigation. A metabolite with a VIP score greater than or equal to 1.0 was considered a variable that highly contributed to the observed separation within the PLS-DA plot. Further description of the extraction protocol, the UPLC-HRMS procedures, and the data processing methods can be found in the Supplemental Information.

### 2.4 Statistical analysis

Data were analyzed using one-way ANOVAs followed by Tukey's post-hocs, Student's t-tests, and chi-square tests, where appropriate. Statistical significance was set at α = 0.05. Data are reported as mean ± SEM, except where noted. Statistical reduction of USFs was accomplished using Student's t-tests (p ≤ 0.05) and VIP score (≥1.0), and individual metabolites of interest were confirmed to be significant through one-way ANOVA analysis and Tukey's post hoc tests.

## 3 Results

### 3.1 Behavioral data

To characterize mice as susceptible or resistant to the effects of acute social defeat stress, mice were evaluated in a social interaction test. One-way ANOVA analysis revealed significant differences in interaction ratios between non-defeated controls, susceptible, and resistant mice (*F*_(2, 30)_ = 31.11, *p* < 0.0001). Tukey's post-hoc comparisons demonstrated that susceptible mice had a significantly lower social interaction ratio compared with resistant mice and controls (p < 0.001 and p < 0.001, respectively), whereas resistant mice and controls did not significantly differ in their social interaction ratios (p = 0.70) (Fig. S-3). A subset of the defeats were quantified, and there were no significant differences between susceptible (8.81 ± 0.74) and resistant (8.22 ± 0.40) mice in the number of attacks received per 2-min e (*t* = 0.38, *df* = 13, *p* = 0.71). No mouse, resistant or susceptible, fought back against the resident aggressor during any of the defeat episodes, and all attacks occurred within the first 30-sec.

In hamsters, the daily dyadic encounters were videotaped and monitored in real time to determine the direction of aggression. On average, dominance relationships were established on day 1.07 (SD = 0.26). Dominant animals continued to direct aggression towards the subordinate throughout the two-week period. Two pairs were excluded because they did not form a dominance relationship after 5 days of dyadic encounters, and one pair was excluded because of a switch in dominance status on day 6. The number of attacks received during social defeat stress, as well as the duration of fighting back by subjects during the first defeat were scored by an observer blind to treatment conditions. There were no significant differences between dominant (5.94 ± 0.86) and subordinate (5.14 ± 0.69) hamsters in the number of attacks received (*t* = 0.91, *df* = 70, *p* = 0.37). Dominant hamsters (8/12) also fought back significantly more often than subordinates (0/12) (χ^2^ = 12.00, *df* = 1, *p* = 0.0005).

### 3.2 Metabolomics data

#### 3.2.1 PLS-DA reveals distinct metabolic profiles in both mice and hamsters

**Fig. 1:**
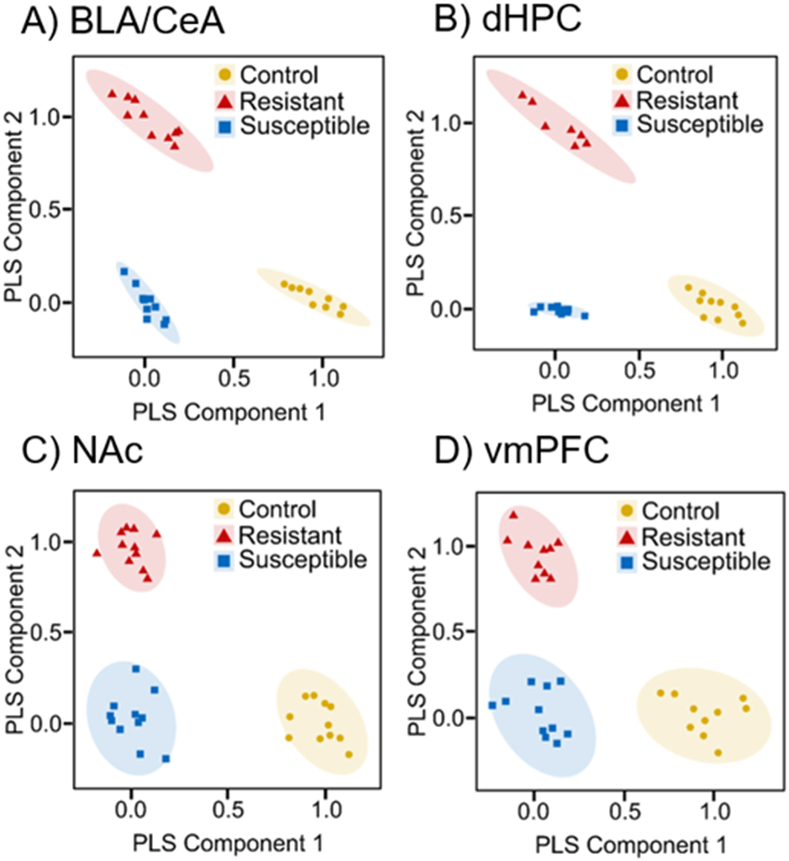
Metabolite patterns from untargeted metabolomic approach. Qualitative profiling for control (yellow), resistant (red), and susceptible (blue) mice was conducted with PLS-DA. PLS component 1 (*X*-axis) and PLS component 2 (*Y*-axis) represent the highest two *X*-scores of dimensions 1 and 2 for matrix *X* of metabolite ion abundances, respectively. Ellipses express a 95% confidence interval. Plots exhibit significant separation and clustering of samples across all four brain regions. A) BLA/CeA, B) dHPC, C) NAc, D) vmPFC. (For interpretation of the references to colour in this figure legend, the reader is referred to the web version of this article.)

**Fig. 2:**
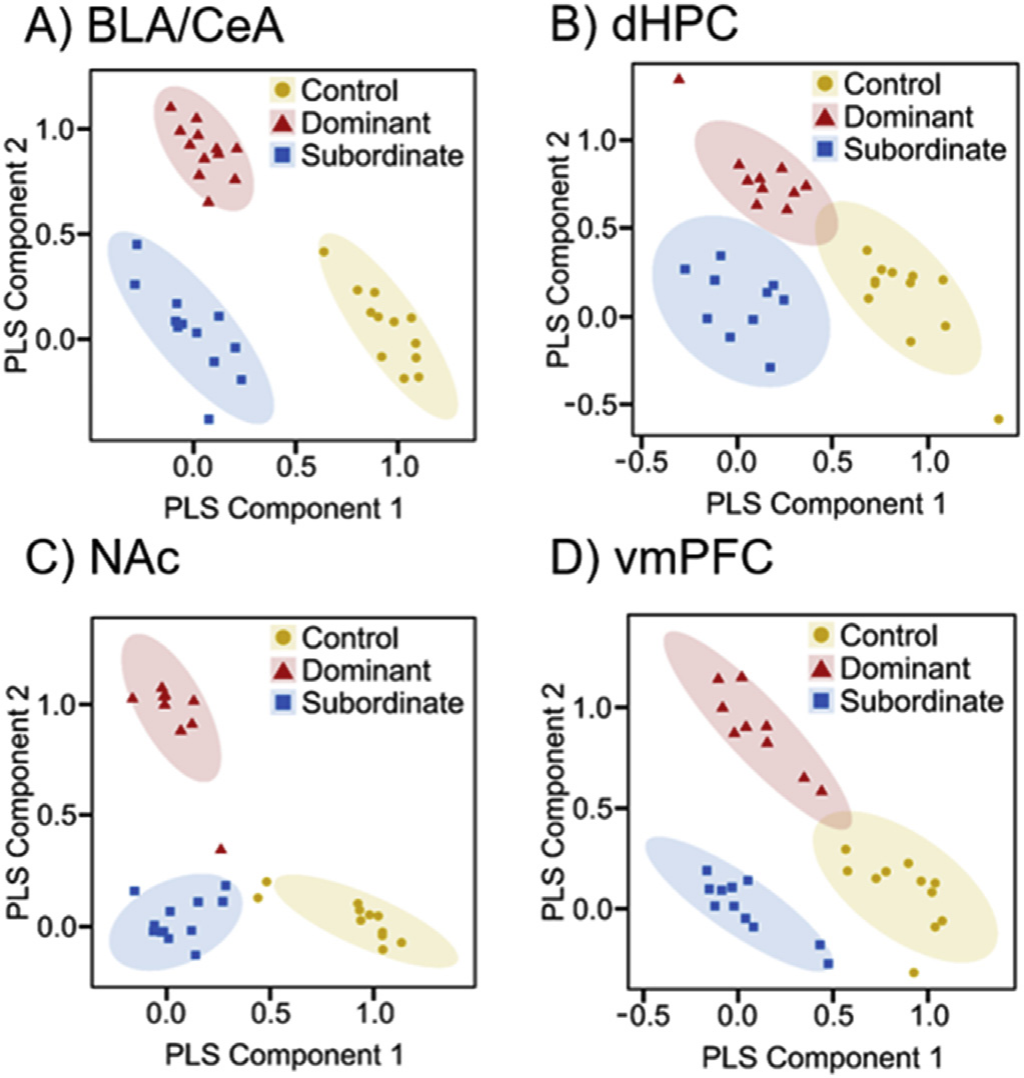
Metabolite patterns from untargeted metabolomic approach. Qualitative profiling for control (yellow), dominant (red), and subordinate (blue) hamsters was conducted with PLS-DA. PLS component 1 (*X*-axis) and PLS component 2 (*Y*-axis) represent the highest two *X*-scores of dimensions 1 and 2 for matrix *X* of metabolite ion abundances, respectively. Ellipses express a 95% confidence interval. Plots exhibit significant separation and clustering of samples across all four brain regions. A) BLA/CeA, B) dHPC, C) NAc, D) vmPFC. (For interpretation of the references to colour in this figure legend, the reader is referred to the web version of this article.)

Fig. 1, Fig. 2 represent the PLS-DA plots generated from the four select brain regions of both mice and hamsters, respectively. The shaded ellipses represent the 95% confidence intervals for each treatment condition. Significant separation of metabolites was achieved for both mice and hamsters, which suggests that the phenotypes exhibit a significantly different metabolic pattern of known and unknown metabolites in each brain region. Furthermore, from the VIP scores derived from the PLS-DA analyses, we extracted variables that differentiated susceptible and resistant mice or subordinate and dominant hamsters. Each brain region exhibited a unique set of relevant (VIP ≥ 1.0) metabolites (Mice; BLA/CeA: 29, dHPC: 25, vmPFC: 33, NAc: 33 and Hamster; BLA/CeA: 48, dHPC: 39, vmPFC: 29, NAc: 29). VIP score plots for both mice and hamsters are available as Supplemental Information (for mice see Fig. S-4, for hamsters see Fig. S-5).

#### 3.2.2 Identifying representative known metabolites

**Fig. 3:**
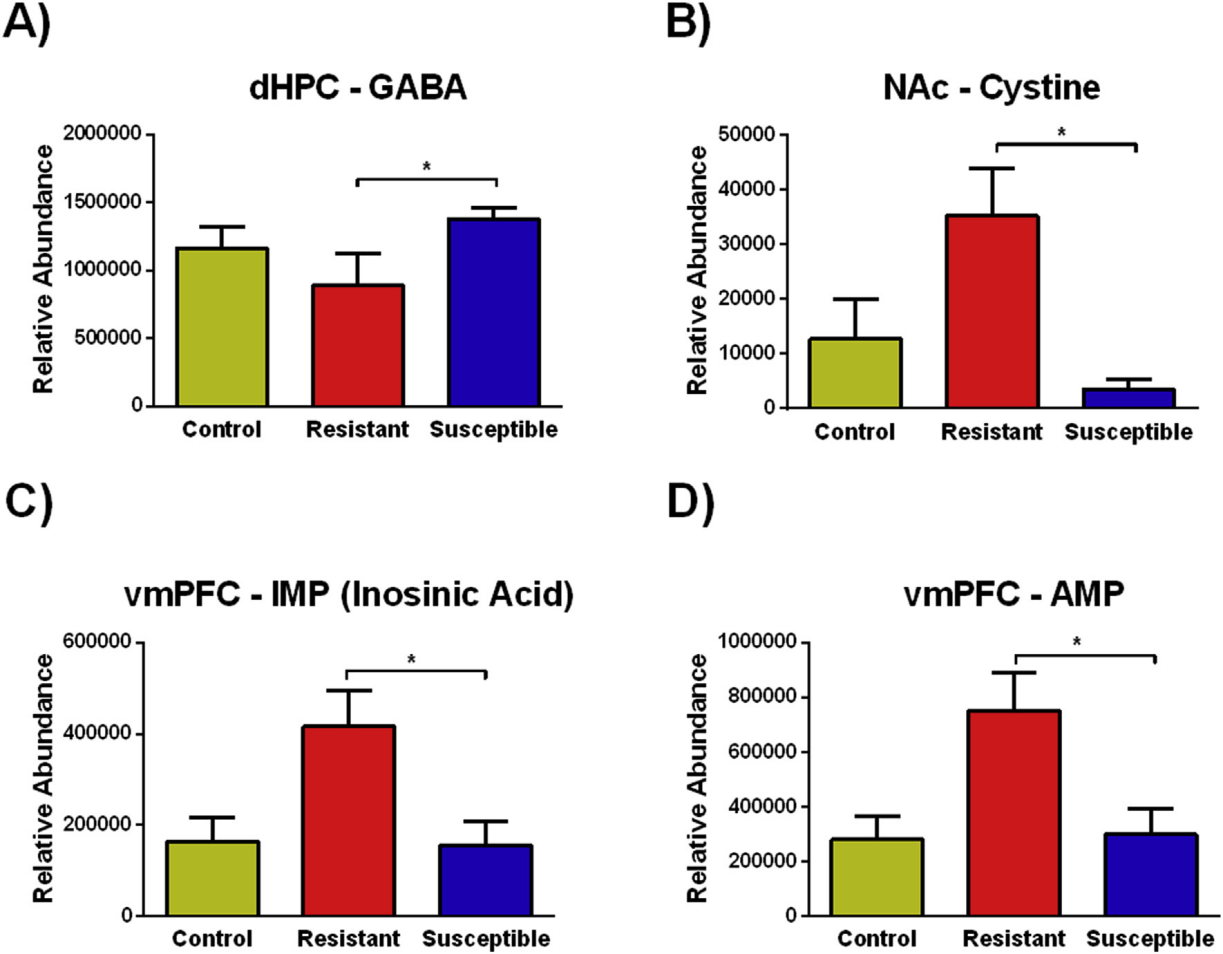
Effects of social defeat on the relative abundance of neurochemical metabolites in mice. A) In the dHPC, susceptible mice had a greater relative abundance of GABA. B) In the NAc, resistant mice had significantly higher levels of cystine. C) In the vmPFC, resistant mice had higher levels of inosinic acid (IMP). D) Finally, also in the vmPFC, resistant mice had a greater relative abundance of AMP. Data are shown as mean ± SEM. An asterisk indicates a significant difference between groups as determined by a Tukey's post hoc test (*p ≤ 0.05).

Representative metabolites were chosen based on the following parameters: *p* ≤ 0.05 and VIP ≥1.0. For mice, one-way ANOVA analyses revealed several significant neurochemical metabolites that differentiated resistant and susceptible animals. In the dHPC, significant differences were observed in the relative abundance of GABA (*F*_(2, 25__)_ = 4.360, *p* = 0.024); specifically, Tukey's post hoc comparisons demonstrated that susceptible mice had elevated levels of GABA compared to resistant mice (Fig. 3A; *p* = 0.018). In the NAc, significant differences were observed in the relative abundance of cystine (*F*_(2, 30)_ = 9.050, *p* = 0.001), and resistant mice had significantly higher levels compared to subordinates (Fig. 3B; *p* = 0.001). In the vmPFC, significant differences were seen in the relative abundance of IMP (inosinic acid) (*F*_(2, 28__)_ = 3.487, *p* = 0.044) and AMP (*F*_(2, 28__)_ = 3.334, *p* = 0.050). For both IMP and AMP, resistant mice had significantly higher levels compared to subordinates (Fig. 3C; *p* = 0.037 and Fig. 3D; *p* = 0.042, respectively). Surprisingly, no significant differences were observed in the BLA/CeA of mice.

**Fig. 4:**
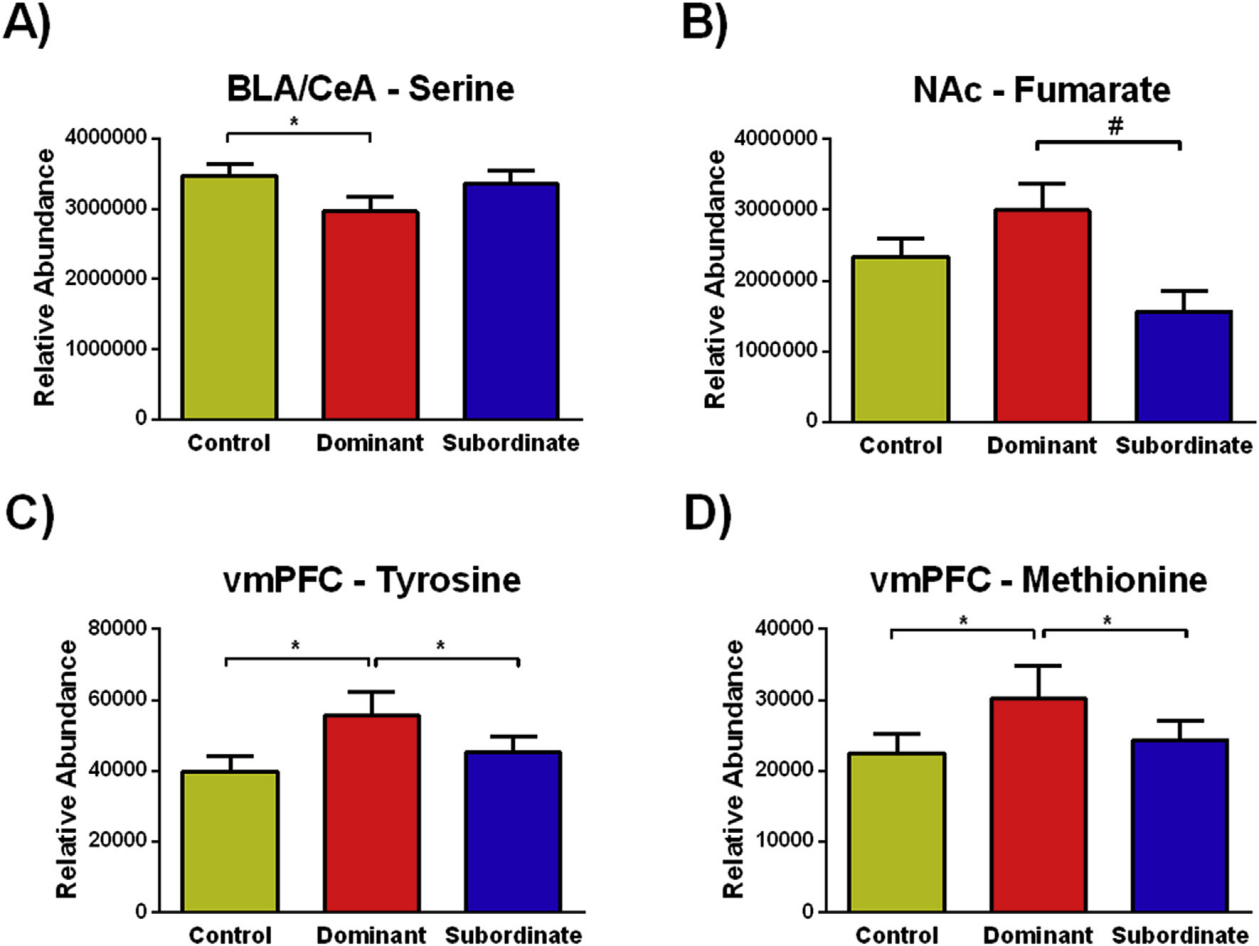
Effects of social defeat and dominance status on the relative abundance of neurochemical metabolites in hamsters. A) In the BLA/CeA, dominant hamsters had significantly lower levels of serine compared to controls. B) In the NAc, dominant hamsters tended to have higher levels of fumarate. C) In the vmPFC, dominant hamsters had a greater relative abundance of tyrosine compared to controls and subordinates. D) Finally, also in the vmPFC, dominant hamsters had significantly higher levels of methionine compared to controls and subordinates. Data are shown as mean ± SEM. An asterisk indicates a significant difference between groups as determined by a Tukey's post hoc test (*p ≤ 0.05, #p = 0.074).

In hamsters, one-way ANOVA analyses also revealed changes in neurochemical metabolite expression. In the BLA/CeA, serine differed significantly across conditions (*F*_(2, 33__)_ = 3.542, *p* = 0.040); specifically, dominants had significantly lower levels compared to controls (Fig. 4A; *p* = 0.034). In the NAc of hamsters, a statistical trend was observed in the relative abundance of fumarate (*F*_(2, 30__)_ = 2.837, *p* = 0.074; VIP = 0.831), and dominant animals tended to have a higher expression compared to subordinates (Fig. 4B; *p* = 0.063). In the vmPFC, tyrosine differed significantly across animals (*F*_(2, 30__)_ = 9.096, *p* = 0.001), and dominant hamsters had a higher relative abundance compared to controls and subordinates (Fig. 4C; *p* = 0.001 and *p* = 0.018, respectively). Also in the vmPFC, significant differences were observed in the expression of methionine (*F*_(2, 30__)_ = 8.017, *p* = 0.002), specifically, dominants had a higher relative abundance compared to controls and subordinates (Fig. 4D; *p* = 0.001 and *p* = 0.023, respectively). However, in the dHPC, no significant differences were observed across conditions.

#### 3.2.3 Identifying representative unknown metabolites

The discovery of thousands of USFs reveals the true complexity of mammalian metabolism. In the BLA/CeA, dHPC, NAc, and vmPFC of hamsters, the total USFs identified were 6981, 5622, 9565, and 7420, respectively, and 2383, 2457, 1987, and 2098 in mice (Table S-1). Following statistical reduction of USFs (see Supplemental Information), we identified a total of 27, 28, 33, and 28 features in hamsters and 27, 27, 30, and 29 features in mice from the BLA/CeA, dHPC, NAc, and vmPFC, respectively. The compilation of USFs represents a list of unique parent masses not found in our list of known metabolites.

Regarding unknown metabolites, only USFs that revealed significant changes between dominant and subordinate hamsters, or resistant and susceptible mice, were further investigated using the Human Metabolome Database (HMDB) (Wishart et al., 2013) to produce a list of potential compound matches. Significance was established through the following parameters: Student t-test: *p* ≤ 0.05, VIP ≥1.0, and the mean decrease in accuracy (MDA) (top 10%). Since each parent mass could exist as an adduct (i.e. [M+Na-H]), the number of matches were anywhere from 0 to 50. Compound matches were screened against existing literature to determine biologically relevant matches.

The following features were selected from both mice and hamsters (Supplemental Information, Table S-2). Two features from the BLA/CeA, m/z 279.0458 (*F*_(2, 26__)_ = 4.919, *p* = 0.015) and m/z 297.0563 (*F*_(2, 26__)_ = 5.2, *p* = 0.013), were significantly reduced in resistant mice compared to controls (*p* = 0.036 and *p* = 0.043, respectively) and susceptible mice (*p* = 0.027 and *p* = 0.017, respectively). These features are believed to be the same compound but detected as separate adducts. In the NAc, the m/z 274.1045 feature (*F*_(2, 30__)_ = 242.613, *p* < 0.001) revealed a significant decrease in susceptible mice compared to controls (*p* < 0.001) and resistant mice (*p* < 0.001), while resistant mice were also significantly increased compared to controls (*p* < 0.001).

In the BLA/CeA of hamsters, the m/z 316.1012 feature (*F*_(2, 33__)_ = 11.073, *p* < 0.001) was significantly increased in subordinates compared to controls (*p* = 0.001) and dominants (*p* < 0.001). Also, in the BLA/CeA, the m/z 289.0903 feature (*F*_(2, 33__)_ = 3.434, *p* = 0.044) was significantly decreased in subordinates compared to dominants (*p* = 0.044). Two features; m/z 320.0619 (*F*_(2, 29__)_ = 3.163, *p* = 0.057) and m/z 342.0441 (*F*_(2, 29__)_ = 5.402, *p* = 0.010) in the NAc were selected as potential adduct masses, which means that they are derived from the same compound. These features showed a decreased concentration in subordinate hamsters compared to dominant hamsters (*p* = 0.050 and *p* = 0.010, respectively).

## 4 Discussion

### 4.1 Identifying the characteristic metabolome associated with social defeat

For the first time, UPLC-HRMS based metabolomics analyses were used to investigate the metabolic fluctuations associated with stress resistance. Metabolomics as a field is currently at its infancy. Complications arising from biological diversity and poor understanding of how to interpret results defines the current state of the field. There are thousands of metabolites within a biological system, not to mention that each metabolite can be involved within multiple metabolic pathways. These current issues present an opportunity to discover novel aspects of biological systems. An untargeted method was used for the current analysis, with the goal of identifying features that could lead to the discovery of a novel biomarker. Specifically, we used a comparative approach to identify similar metabolites that are associated with stress resistance in both models. The varying levels of global metabolites for both mice and hamsters were analyzed using PLS-DA, which resulted in significant clustering of subjects and separation of phenotypes. The separation of the phenotypes in the PLS-DA plots suggests that stress-induced metabolic fluctuations can reliably predict phenotypic responses to social defeat stress. Based on VIP scores and Tukey's post hoc comparisons, significant fluctuations were found in amino acid and neurotransmitter metabolism that are associated with stress susceptibility and resistance in both mice and hamsters.

Interestingly, both mice and hamsters that are susceptible and resistant to social defeat stress show changes in small molecules in the NAc that modulate oxidative stress. The relative abundance of the amino acid cystine was higher in resistant mice compared to susceptible mice. Cystine is a critical component of the cystine-glutamate exchange, also known as the system xc–. The uptake of cystine that results from a cystine-glutamate exchange is important for maintaining the levels of glutathione, a critical antioxidant (Bridges et al., 2012, Reissner, 2014). Furthermore, system xc-dependent glutathione production modulates protection from oxidative stress (Shih et al., 2006). Taken together, the upregulation of cystine in resistant mice may help protect them against defeat-induced social avoidance. In hamsters, the levels of fumarate tended to be higher in the NAc of dominants compared to subordinates. Fumarate is an intermediate in the Krebs cycle used by cells to produce energy in the form of adenosine triphosphate (ATP). Not surprisingly, oxidative stress suppresses the Krebs cycle (Tretter and Adam-Vizi, 2000). Esterification of the unsaturated dicarbonic acid, fumarate can be enzymatically catalyzed to form fumaric acid esters (FAEs), such as dimethyl fumarate that has been shown to exert neuroprotective effects against neuroinflammation via activation of the Nrf2 antioxidant pathway (Linker et al., 2011). Additionally, research has also shown that dimethyl fumarate protects cells from oxidative stress (Albrecht et al., 2012). In addition to these molecules within the NAc that modulate oxidative stress, evidence of protection from oxidative stress was further observed in the vmPFC of dominant hamsters. To illustrate, methionine was elevated in dominants compared to both controls and subordinates. Methionine is an antioxidant that has been shown to reverse the effects of oxidative stress (Patra et al., 2001, Nandi et al., 2005, Luo and Levine, 2009). Overall, subjects resistant to acute social defeat had elevated levels of small molecules in the NAc and vmPFC following social defeat that protect against oxidative stress, such as cystine, fumarate, and methionine.

This study also revealed significant defeat-induced changes in neurotransmitters and their precursors. In the dHPC, susceptible mice had significantly higher levels of GABA compared to both resistant and control mice. Stress exposure has been shown to increase GABA levels in the hippocampus. For instance, mild stressors such as novel cage exposure and more intense stressors such as forced swimming both increase hippocampal GABA levels (De Groote and Linthorst, 2007). Furthermore, microdialysis reveals increased extracellular levels of GABA in response to novelty stress in the vHPC (Bianchi et al., 2003). In hamsters, we found that dominant animals had increased tyrosine concentrations in the vmPFC compared to subordinates. Tyrosine serves as a critical precursor to catecholamines and has antioxidant properties (Yen and Hsieh, 1997, Gülçin, 2007). Rats given tyrosine before acute tail-shock displayed neither shock induced norepinephrine depletion nor the deficits in exploratory behaviors observed in saline-treated animals (Reinstein et al., 1984). Similarly, pre-treatment with tyrosine not only prevented behavioral depression and norepinephrine depletion after acute restraint stress and intermittent tail-shock but also suppressed the rise in plasma corticosterone (Reinstein et al., 1985).

Additionally, we found that dominant hamsters had lower serine concentrations in the BLA/CeA compared to control hamsters. D-serine is an endogenous modulator of the glycine site of NMDA receptors and functions to facilitate memory processes (Mothet et al., 2000, Collingridge et al., 2013). Systemic administration of D-serine has been shown to enhance both object recognition and T-maze performance (Bado et al., 2011). While partial agonists at the glycine site on NMDA receptors have been proposed as cognitive enhancers to facilitate cognitive behavioral therapy (Davis et al., 2006), systemic administration of D-serine induces oxidative stress in the rat brain (Armagan et al., 2011). One possibility is that dominant animals may have less endogenous serine to bind to the glycine site of the NMDA receptor in the BLA, which may also reduce the potential for oxidative stress.

Finally, in the vmPFC of mice, changes were observed in molecules relate to cellular energy consumption. More specifically, resistant mice had significantly higher levels of IMP (inosinic acid) and AMP (adenosine monophosphate) in the vmPFC immediately following social defeat compared to susceptible mice. AMP is produced during adenosine triphosphate (ATP) synthesis, while IMP is generated via ATP degradation in two metabolic pathways in which AMP is generated and then degraded to either IMP or adenosine (Seki et al., 2017). Taken together, these two molecules are indicative of increased cellular activity in the vmPFC of resistant mice.

### 4.2 Characterization of unidentified spectral features affected by social stress

The current state of metabolomics research is focused on the structural elucidation of USFs from an untargeted experiment. Through this technology, the breadth of the metabolome can be revealed but there are many steps involved with confirmation of structure. This hypothesis driven experiment has produced thousands of USFs, which are potential metabolites that could better explain the entire metabolome of a subject. Here, we provided a high-throughput method for the characterization of USFs. In this experiment, the aim was to find a reduced list of metabolites that exhibited significant metabolic changes associated with social stress defeat and provide possible compound matches to be evaluated in future experiments.

Preliminary annotation of USFs revealed a common trend in which several of the compounds were related to oxidative stress. In the NAc of mice, the 274.1945 m/z at 4.7 min feature was matched to norophthalmic acid. This neurochemical was significantly decreased in susceptible mice and increased in resistant mice. As a tripeptide analogue of glutathione, norophthalmic acid has been shown to be an intracellular antioxidant. It is known that disturbance of glutathione homeostasis either leads to or results from oxidative stress (Gonçalves et al., 2008, Schulz et al., 2000). This was particularly interesting because cystine levels in NAc of mice were also elevated in resistant mice after social defeat. As a dimer of cysteine, fluctuations in concentrations of this compound have a direct effect on levels of glutathione. Because resistant mice show increased concentrations of both compounds, it suggests that susceptible mice respond to social defeat stress with fewer essential antioxidants which may reduce their coping ability. Also, in the BLA/CeA of hamsters, the 289.0903 m/z at 11.6 min feature corresponded to N-acetylcarnosine (NAC). This free-radical scavenger is a natural N-acetylated dipeptide consisting of alanine and histidine. Carnosine derivatives have been shown to act as natural antioxidants with hydroxy radical, singlet oxygen scavenging and lipid peroxidase properties (Babizhayev et al., 1994). Although this compound hasn't been associated with stress-related behavior, NAC treatment has been shown to reduce the effects of cataracts, which are caused by oxidative stress on the lens (Babizhayev et al., 2001). In the present study, NAC was increased in dominant hamsters, again suggesting a potential mechanism that protects against oxidative stress.

In a separate correlation, two USF features from hamsters exhibited effects on the efficiency of mitochondrial activity. In mammalian cells, mitochondria have been shown to produce reactive oxygen species (ROSs) to reduce oxidative damage and contribute to redox signaling to the nucleus (Murphy, 2009). L-acetylcarnitine (LAC) has a known biological function of improving the efficiency of mitochondrial function by facilitating the movement of acetyl CoA into the matrices (Wutzke and Lorenz, 2004). In our study, the amount of LAC (316.1012 at 11.6 min) was significantly increased in the BLA/CeA of subordinate hamsters. This observation reveals that the cellular mitochondrial activity is being up-regulated in subordinate hamsters after social defeat. Additionally, the NAc samples revealed a significant decrease of relative concentration in subordinate hamsters for a set of parent masses at 15 min; 320.0619 ([M-H]) and 342.0441 ([M+Na-2H]). These features both correlated to beta-citryl-L-glutamic acid (BCG), which is a derivative of glutamate found in the developing brain of rats (Miyake et al., 1978). This metabolite has been shown to be an iron carrier that is used to activate the enzyme aconitase (Hamada-Kanazawa et al., 2011), which is used to enhance cell viability by accelerating mitochondrial activity. Alterations to normal mitochondrial function during stress can contribute to cell death through two mechanisms; change in production of ROSs and release of death regulatory and signaling molecules from the intermembrane space (García-Ruiz et al., 1995, Ouyang and Giffard, 2004).

Finally, preliminary annotation of unknown metabolites indicates changes in small molecules associated with memory function. For example, in the BLA/CeA of mice, two features (m/z, 279.0458 ([M-H]) and 297.0563 ([M-H2O-H])) at 11.5 min revealed the existence of the same compound, N-acetylserotonin sulfate (NAS). NAS is an intermediate in the endogenous production of melatonin from serotonin (Weissbach et al., 1960), and has been shown to act as a classic antioxidant through hydrogen donation (Barsacchi et al., 1998). NAS has also been shown to improve cognition (Álvarez-Diduk et al., 2015) and increases BDNF levels (Yoo et al., 2011). Interestingly, NAS has been shown to directly activate the TrkB receptor (Jang et al., 2010). TrkB, as the primary receptor for BDNF, is critical for learning and memory related plasticity (Minichiello, 2009). One possibility is that increased NAS in susceptible mice is associated with a stronger memory of the defeat experience. This possibility is consistent with our previous finding that inhibition of the BDNF synthesis pathway in the BLA reduced defeat-induced social avoidance (Dulka et al., 2016).

Some limitations of the present study should be acknowledged. While untargeted metabolomics provides opportunity for the discovery of novel biomarkers, large quantities of data are produced and an agreed upon high-throughput method for structure elucidation has not been validated. We have used statistical significance to reduce the number of USF considered for discussion, although additional tandem MS/MS analyses should be performed to definitively characterize novel metabolites. Additionally, this study is limited in that it only used male subjects, which is particularly concerning given that women are more likely to develop a stress-related mental illness (Kessler, 2003, Bekker and van Mens-Verhulst, 2007). Indeed, a sex bias exists in neuroscience research (Beery and Zucker, 2011), especially within the realm of social defeat models. One noteworthy exception can be seen in work with the California mouse (*Peromyscus californicus*), a monogamous species in which both males and females aggressively defend their home territories (Ribble and Salvioni, 1990). In this species, females are particularly susceptible to social defeat compared to males, and analyses of brain activity immediately following social interaction testing suggested that cellular activity in the NAc may be related to social avoidance (Trainor, 2011). While these data suggest that the NAc may be ideally situated to study biomarkers in females, sex-differences in stress-induced social withdrawal in the California mouse are also associated with BDNF in the bed nucleus of the stria terminalus (Greenberg et al., 2013). Overall, further research is needed to characterize the stress-induced neurochemical profiles of females.

## 5 Conclusions

We found that 18% of C57 mice show resistance to social avoidance following an acute social defeat. Similarly, hamsters that have maintained dominant social status exhibit less defeat-induced social avoidance compared to subordinates. While an analysis of defeat-induced changes in metabolites within select brain regions indicates that the concentrations of many small molecules differ between susceptible and resistant animals, no single biomarker was observed in both species. However, both dominant hamsters and resistant mice show changes in neurochemicals within similar functional classes. Importantly, animals resistant to social defeat stress show an increased concentration of molecules to protect against oxidative stress in the NAc and vmPFC. Overall, a metabolomics approach to the study of stress resilience can identify functional groups of neurochemicals that may serve as novel targets for the treatment of stress-related mental illness.
