# Supplementary material for: Metabolomics reveals distinct neurochemical profiles associated with stress resilience
Source: Neurobiol Stress. 2017 Aug 7;7:103–12. doi: 10.1016/j.ynstr.2017.08.001 (PMC5552108; doi:10.1016/j.ynstr.2017.08.001)
Supplement: Supplementary material [file mmc1.docx]

Metabolomics Reveals Distinct Neurochemical Profiles Associated with Stress Resilience

Supplemental Information

Brooke N. Dulka1†*, Allen K. Bourdon2†, Catherine T. Clinard1, Mohan B.K. Muvvala1, Shawn R. Campagna2,3, & Matthew A. Cooper1

1Department of Psychology

2Department of Chemistry

3Biological Small Molecule Mass Spectrometry Core

University of Tennessee, Knoxville, TN 37996

†These authors contributed equally.

*Correspondence: [bdulka@vols.utk.edu](mailto:bdulka@vols.utk.edu)

**Overview.** This supplemental information contains additional methods and figures from the analysis of social avoidance behavior in mice and the untargeted metabolomics data. The figures include supplemental behavioral data, heatmaps with p-values from complimentary t-test statistics, and variable importance of projection (VIP) score plots derived from partial least squares discriminant analysis (PLS-DA). PLS-DA and VIP figures include data from all four brain regions; basolateral/central amygdala (BLA/CeA), dorsal hippocampus (dHPC), nucleus accumbens (NAc), and ventromedial prefrontal cortex (vmPFC), from both mice and hamster subjects. Additionally, a comprehensive table includes the total number of unidentified spectral features (USFs) detected and how statistical reduction can reduce the overall complexity of the data. Lastly, a table is included for the selected USFs for further discussion in the manuscript.

**METHODS AND MATERIALS**

*Untargeted Metabolomics*

*Extraction Protocol*

Samples were transferred from a -80°C freezer to a 5°C cold room, where they were left to thaw for 20 minutes. The samples were then extracted using a modified acidic acetonitrile protocol (Rabinowitz and Kimball 2007). To the tissue punches, 500μL of extraction solvent (40:40:20 acetonitrile/methanol/water containing 0.1 M formic acid) was added and the mixture was transferred to -20° C for 20 minutes. The samples were then centrifuged for 5 minutes at 13,000 rpm and the resulting supernatant was collected. Pellets were washed twice with 100 μL aliquots of extraction solvent. The combined supernatants were then dried under nitrogen, followed by resuspension with 150μL of Millipore water.

*Ultra-High Performance Liquid Chromatography-High-Resolution Mass Spectrometry (UPLC-HRMS)*

Analysis by UPLC-HRMS was completed using an ultimate 3000 LC pump coupled with an Exactive Plus Orbitrap MS (Thermo Fisher Scientific, Waltham, MA). All solvents were HPLC grade and purchased from Fisher Scientific (Atlanta, GA). The mobile phases, solvent A (97:3 water:methanol containing 10 mM tributylamine, and 15 mM acetic acid) and solvent B (methanol), were set to use a multi-step gradient as follows; 0 to 5 min, 0% B; 5 to 13 min, 20% B; 13 to 15.5 min, 55% B; 15.5 to 19 min, 95% B; and 19 to 25 min, 0% B. With a flow rate of 200 μL/min, a 10 μL aliquot of sample was injected through a Synergi 2.5 μm Hydro-RP 100, 100 x 2.0 mm LC column (Phenomenex) maintained at 25°C. While running full scan in negative mode, the eluent was introduced to the MS by an electrospray ionization source (Lu et al. 2010). Samples were analyzed with a resolving power of 140,000 and a scan window of 72 to 800 *m/z* from 0 to 9 min, and 100 to 1,000 *m/z* from 9 to 25 min. Samples were injected in a randomized order to reduce instrumental error.

*Data Processing*

Raw files generated by the Xcaliber software (Thermo Fisher Scientific) were converted to an open source format, mxML (Martens et al. 2011), via the open source MSConvert software, which is part of the ProteoWizard package (Chambers et al. 2012). The converted files were then uploaded into the Metabolomic Analysis and Visualization Engine (MAVEN) software, which automatically adjusts the total ion chromatograms based on retention times for each sample (Melamud, Vastag, and Rabinowitz 2010).

Using MAVEN (Melamud, Vastag, and Rabinowitz 2010), metabolites were selected from a pre-existing list of 270 known compounds with annotated biological activity. All retention times were previously validated for exact mass and retention time using chemical standards from Fisher Scientific or preexisting chromatography (Lu et al. 2010). Integration of the area under the curve provided each metabolite with a total ion count that can be considered the relative concentration of the metabolite within a single sample.

*Internal Ratio Normalization (IRN)*

By assuming that intensity ratios within a sample are consistent across all metabolites, an internal ratio can be used to normalize the data in an attempt to reduce instrumental and technical variability. This technique constructs a correlation type matrix for each sample, which is composed of the individual ratios across every metabolite within the sample. These generated matrices are then averaged among replicates, followed by comparison between treatments. Using the IRN R script (https://irn-access:bu609campagna@gitlab.com/irn/irn.git), the data were normalized to produce fold change and p-values across experimental comparisons. These normalized values were used to produce the heatmap figures for known metabolites. The discussed IRN script is currently in submission for publication.

*Heatmaps*

The fold-change data, after IRN, were log 2 transformed and clustered by average linkage using Cluster software, version 3.0 (Eisen Lab, Berkeley, CA). This data was then displayed in a heatmap generated by Java Treeview software (Saldanha 2004). Using a saturated color scale ranging from +3 (red) to -3 (blue), visual comparison of concentration changes can be observed. The two array comparisons are listed above each column on the heatmap. Each cell has been overlaid with a symbol representing a specific p-value range (≤ 0.01 – “***”; ≤ 0.05 – “**”; ≤ 0.1 – “*”). Heatmap figures can be found in Supplemental Information (For mice see Figure S-2, for hamsters see Figure S-3).

*Partial least squares discriminant analysis (PLS-DA)*

Within the field of metabolomics, a widely accepted statistical tool used to confirm variation among variables is PLS-DA. As a two-dimensional, multivariate linear regression model, PLS-DA finds the direction of maximum covariance between a data set (*X*) and the class (*Y*) (Wold, Sjöström, and Eriksson 2001). The original variables are compressed into fewer variables by using their weighted averages, referred to as scores.

Following compilation of all spectral features detected (includes both identified and unidentified spectral features), the grouped data was loaded into RStudio (Racine 2012). To generate the PLS-DA data, the DiscriMiner package was utilized and specifically the PLS-DA function. All data were normalized by using an auto-scaling method that attempts to scale and center the data by dividing each value by the metabolites standard deviation. By extracting the PLS-DA scores values, the visualization aid was generated using the ggplot package with a 95% confidence interval. Variable importance projection (VIP) score plots were also generated from the plsDA function of DiscriMiner. VIP plots for both mice and hamsters can be found in the Supplemental Materials (for mice see Figure S-4, for hamsters see Figure S-5). These VIP plots only represent the identified spectral features to facilitate interpretation.

*Identification and Statistical Reduction of Unidentified Spectral Features (USFs)*

The MAVEN software includes a peak picking algorithm that generates a list of all spectral features detected by the HRMS in a single sample. A user generated training model establishes a quality score for each peak, which enables the software to identify thousands of USFs from each sample. All peaks were confirmed manually to have a proper Gaussian curve, 3:1 signal-to-blank, and an ion count greater than 103.

Next, the lists were reduced using Student’s t-tests for three separate array comparisons; resistant vs. control, susceptible vs. control, and resistant vs. susceptible for mice or dominant vs. control, subordinate vs. control, and dominant vs. subordinate for hamsters. Using this statistical tool dramatically reduces the overall number of identified metabolites, which are then manually investigated to remove any “known metabolites” that were previously identified, as well as any carbon-13 isomers.

Finally, the web-based statistical analysis tools of Metaboanalyst (Xia et al. 2009) were used to focus on a small number of these USFs. Through cross comparison of the variable importance projection (VIP) score plots generated from the PLS-DA and the mean decrease in accuracy (MDA) plots from the random forest classification, a reduced list of ~30 important USFs were extrapolated and used for additional investigation. By viewing Supplemental Table 1 (Table S-1), the specific quantities of USFs after each step can be observed for each brain region.

*Statistical Analysis*

Data were analyzed using one-way ANOVAs followed by Tukey post-hocs and Student’s t-tests, where appropriate. Statistical significance was set at α = 0.05. Data are reported as mean ± SEM, except where noted. Statistical reduction of USFs was accomplished using Student’s t-tests (p ≤ 0.05) and VIP score (≥ 1.0) and metabolites selected for further discussion were confirmed to be significant through one-way ANOVA analysis and Tukey’s post hoc tests.

**FIGURES**


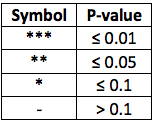

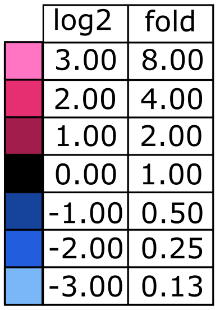

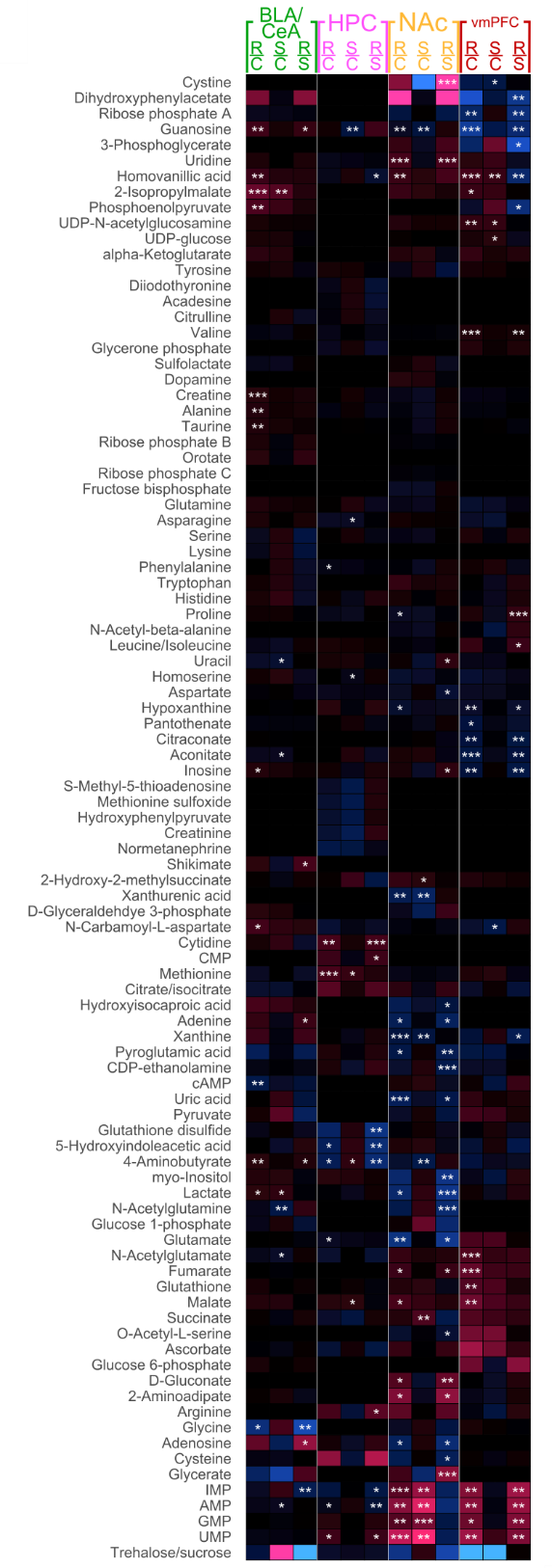


**Figure S-1.** Heatmap of all identified metabolites in mice from 4 brain regions. Array comparisons are labeled at the top of the figure; R/C: resistant/control, S/C: susceptible/control, and R/S: resistant/susceptible (numerator/denominator). Saturated colors are displayed on a log 2 scale. A Student’s t-test was used to produce the p-values using a two-tail comparison of the arrays. Values were converted to symbols and overlaid onto the figure.


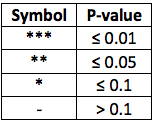

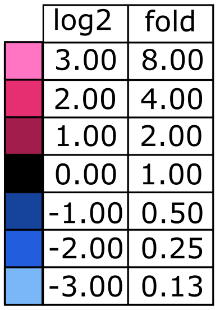

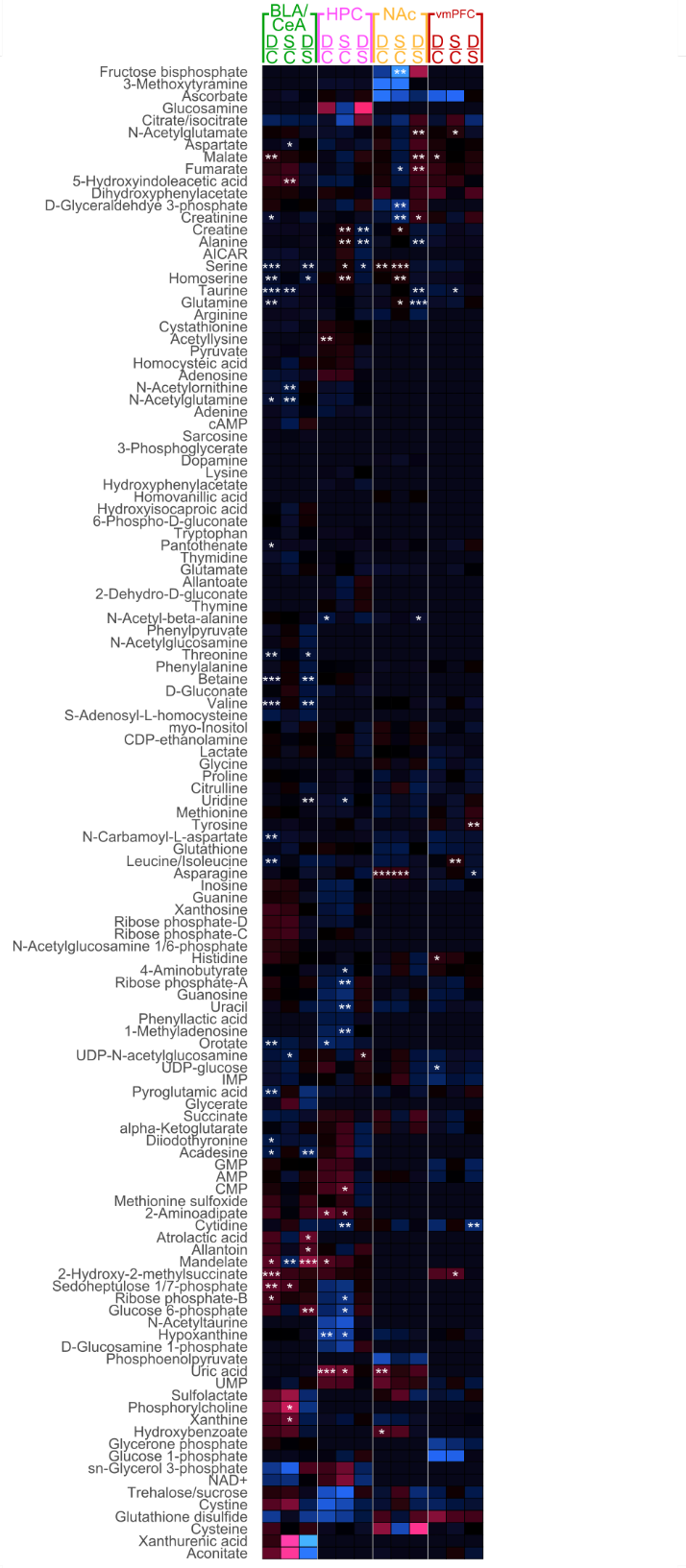


**Figure S-2.** Heatmap of all identified metabolites in hamsters from 4 brain regions. Array comparisons are labeled at the top of the figure; D/C: dominant/control, S/C: subordinate/control, and D/S: dominant/subordinate (numerator/denominator). Saturated colors are displayed on a log 2 scale. A Student’s t-test was used to produce the p-values using a two-tail comparison of the arrays. Values were converted to symbols and overlaid onto the figure.

**
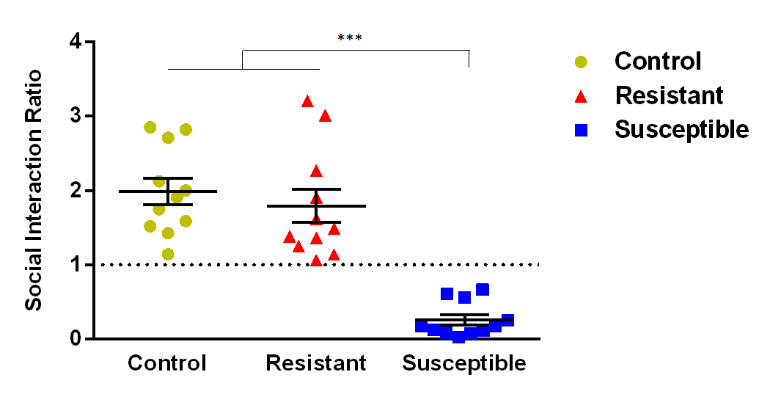
**

**Figure S-3.** Mice were tested in a neutral arena for social avoidance 24 hours after their first social defeat experience. We segregated defeated mice into two populations, resistant and susceptible, based on their social interaction ratios. Mice with a social interaction ratio of 1 or greater were characterized as resistant, while mice with a social interaction ration of less than 1 were characterized as susceptible. All non-defeated control mice always had a social interaction ration greater than 1. One-way ANOVA analysis revealed significant differences in interaction ratios between non-defeated controls, susceptible, and resistant mice (F(2, 30) = 31.11, p < 0.0001). Tukey’s post-hoc comparisons demonstrated that susceptible mice had a significantly lower social interaction ratio compared with resistant mice and controls (p < 0.001 and p < 0.001, respectively), whereas resistant mice and controls did not differ in their social interaction ratios (p = 0.70).


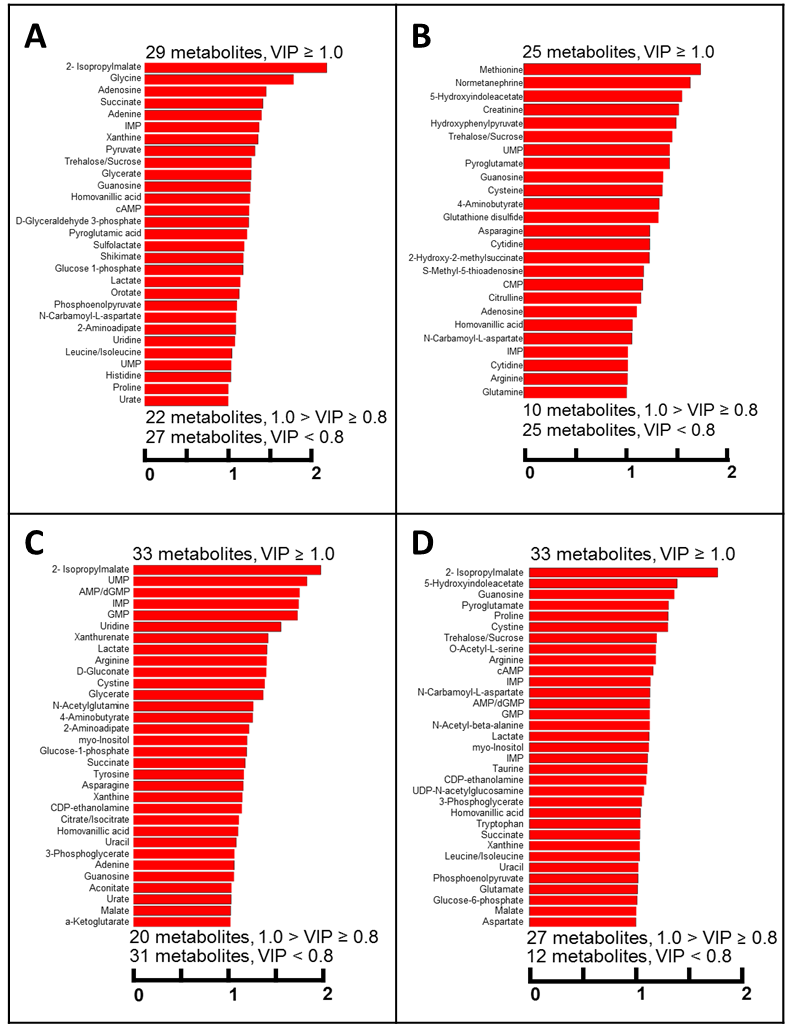


**Figure S-4.** VIP plots of identified metabolites in mice with a VIP score ≥ 1.0. Labels are provided to explain the total number of metabolites within three ranges; VIP ≥ 1.0, 1.0 < VIP ≥ 0.8, and VIP < 0.8. These plots are derived from PLS-DA multivariate analysis. A) BLA/CeA, B) dHPC, C) NAc, D) vmPFC.


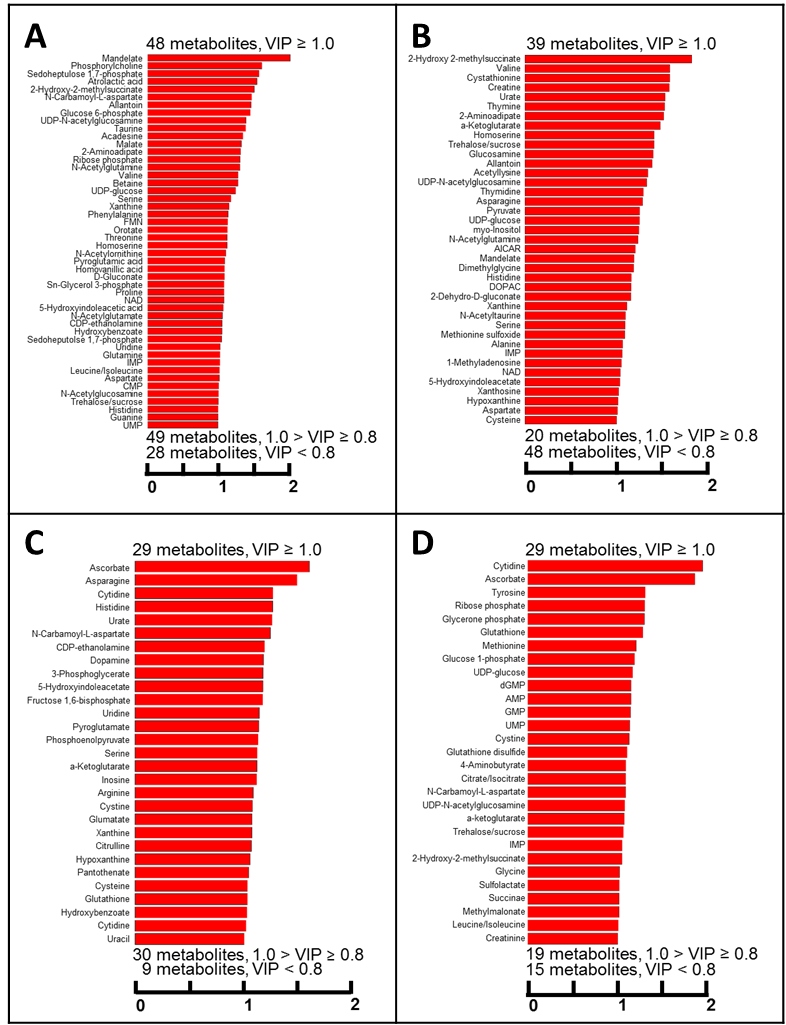


**Figure S-5.** VIP plots of identified metabolites in hamsters with a VIP score ≥ 1.0. Labels are provided to explain the total number of metabolites within three ranges; VIP ≥ 1.0, 1.0 < VIP ≥ 0.8, and VIP < 0.8. These plots are derived from PLS-DA multivariate analysis. A) BLA/CeA, B) dHPC, C) NAc, D) vmPFC.

**TABLES**

**Table S-1.** Total number of unidentified spectral features (USFs) detected using UPLC-HRMS for untargeted metabolomics.

*Significant features refers to p-value ≤ 0.05 (in a Student’s t-test), variable importance in projection (VIP) ≥ 1.0, and the mean decrease in accuracy (MDA) = top 10%.

All raw data will be made available upon request to the Campagna Lab, University of Tennessee, Knoxville.

**Table S-2.** Unidentified spectral features (USFs) selected for discussion. Statistical reduction using Student’s t-tests identified several USFs with Human Metabolome Database (HMDB) matches (Wishart et al. 2013), which were then confirmed to be significant through one-way ANOVA analysis and Tukey’s post-hoc tests.

**References**

Chambers, M.C., Maclean, B., Burke, R., Amodei, D., Ruderman, D.L., Neumann, S., Gatto, L., Fischer, B., Pratt, B. and Egertson, J. 2012. A cross-platform toolkit for mass spectrometry and proteomics. Nature biotechnology, 30: 918-920.

Lu, W., Clasquin, M.F., Melamud, E., Amador-Noguez, D., Caudy, A.A. and Rabinowitz, J.D. 2010. Metabolomic Analysis via Reversed-Phase Ion-Pairing Liquid Chromatography Coupled to a Stand Alone Orbitrap Mass Spectrometer. Analytical Chemistry, 82: 3212-3221.

Martens, L., Chambers, M., Sturm, M., Kessner, D., Levander, F., Shofstahl, J., Tang, W.H., Römpp, A., Neumann, S. and Pizarro, A.D. 2011. mzML—a community standard for mass spectrometry data. Molecular & Cellular Proteomics, 10: R110. 000133.

Melamud, E., Vastag, L. and Rabinowitz, J.D. 2010. Metabolomic analysis and visualization engine for LC− MS data. Analytical chemistry, 82: 9818-9826.

Rabinowitz, J.D. and Kimball, E. 2007. Acidic Acetonitrile for Cellular Metabolome Extraction from Escherichia coli. Analytical Chemistry, 79: 6167-6173.

Racine, J.S. 2012. RStudio: A Platform-Independent IDE for R and Sweave. Journal of Applied Econometrics, 27: 167-172.

Saldanha, A.J. 2004. Java Treeview—extensible visualization of microarray data. Bioinformatics, 20: 3246-3248.

Wishart, D.S., Jewison, T., Guo, A.C., Wilson, M., Knox, C., Liu, Y., Djoumbou, Y., Mandal, R., Aziat, F., Dong, E., Bouatra, S., Sinelnikov, I., Arndt, D., Xia, J., Liu, P., Yallou, F., Bjorndahl, T., Perez-Pineiro, R., Eisner, R., Allen, F., Neveu, V., Greiner, R. and Scalbert, A. 2013. HMDB 3.0—The Human Metabolome Database in 2013. Nucleic Acids Research, 41: D801-D807.

Wold, S., Sjöström, M. and Eriksson, L. 2001. PLS-regression: a basic tool of chemometrics. Chemometrics and intelligent laboratory systems, 58: 109-130.

Xia, J., Psychogios, N., Young, N. and Wishart, D.S. 2009. MetaboAnalyst: a web server for metabolomic data analysis and interpretation. Nucleic Acids Research, 37: W652-W660.
